# Advancing maturity modeling for precision oncology

**DOI:** 10.1017/cts.2023.682

**Published:** 2023-12-07

**Authors:** Ariella Hoffman-Peterson, Megh Marathe, Mark S. Ackerman, William Barnett, Reema Hamasha, April Kang, Kashmira Sawant, Allen Flynn, Jodyn E. Platt

**Affiliations:** 1 University of Michigan, Ann Arbor, MI, USA; 2 Michigan State University, East Lansing, MI, USA; 3 Harvard Medical School, Boston, MA, USA

**Keywords:** Precision oncology, maturity models, learning health systems, learning cycle, molecular tumor boards

## Abstract

**Introduction:**

This study aimed to map the maturity of precision oncology as an example of a Learning Health System by understanding the current state of practice, tools and informatics, and barriers and facilitators of maturity.

**Methods:**

We conducted semi-structured interviews with 34 professionals (e.g., clinicians, pathologists, and program managers) involved in Molecular Tumor Boards (MTBs). Interviewees were recruited through outreach at 3 large academic medical centers (AMCs) (*n* = 16) and a Next Generation Sequencing (NGS) company (*n* = 18). Interviewees were asked about their roles and relationships with MTBs, processes and tools used, and institutional practices. The interviews were then coded and analyzed to understand the variation in maturity across the evolving field of precision oncology.

**Results:**

The findings provide insight into the present level of maturity in the precision oncology field, including the state of tooling and informatics within the same domain, the effects of the critical environment on overall maturity, and prospective approaches to enhance maturity of the field. We found that maturity is relatively low, but continuing to evolve, across these dimensions due to the resource-intensive and complex sociotechnical infrastructure required to advance maturity of the field and to fully close learning loops.

**Conclusion:**

Our findings advance the field by defining and contextualizing the current state of maturity and potential future strategies for advancing precision oncology, providing a framework to examine how learning health systems mature, and furthering the development of maturity models with new evidence.

## Introduction

**Figure 1.**
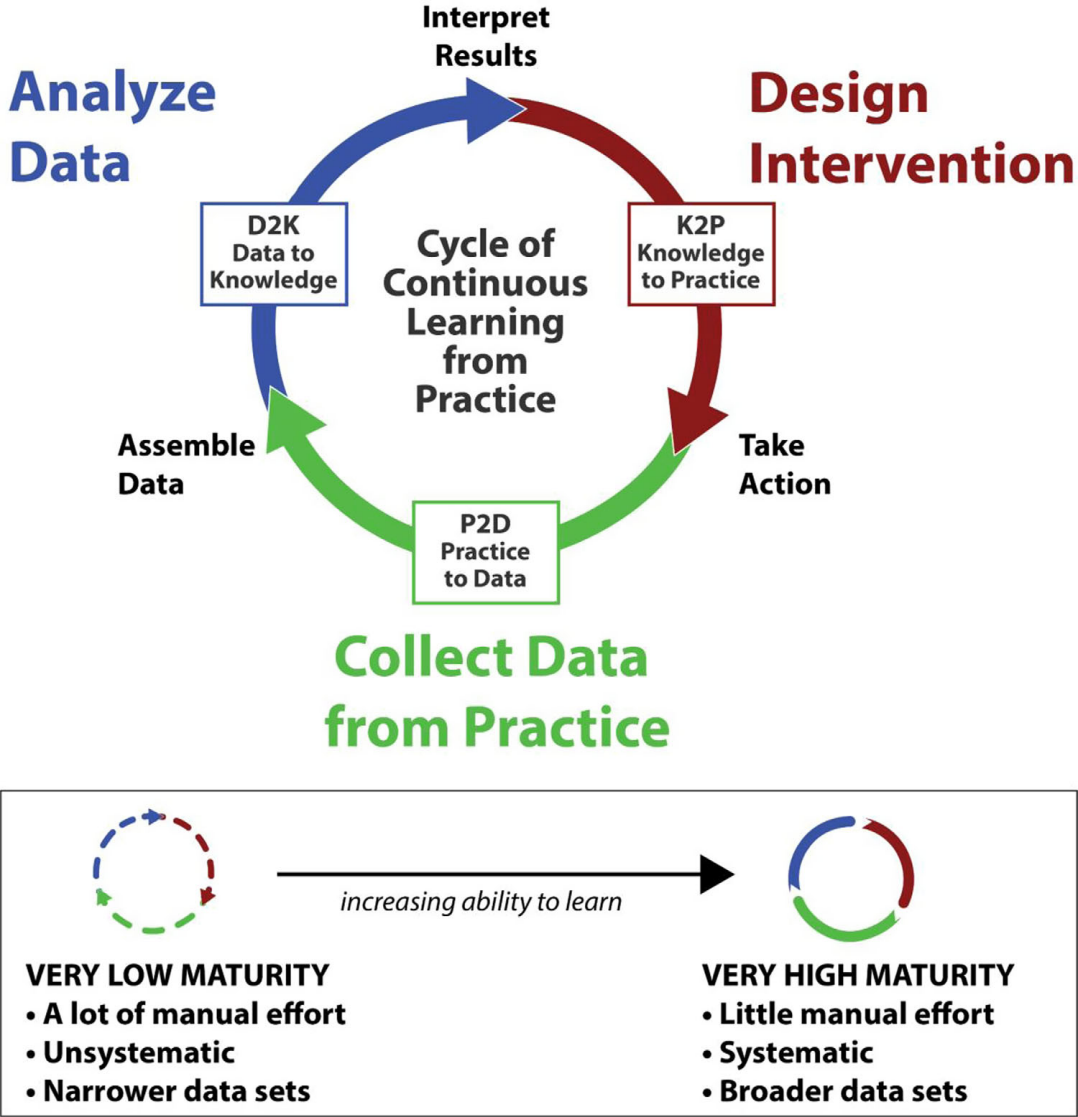
Learning cycle [2].

**Figure 2.**
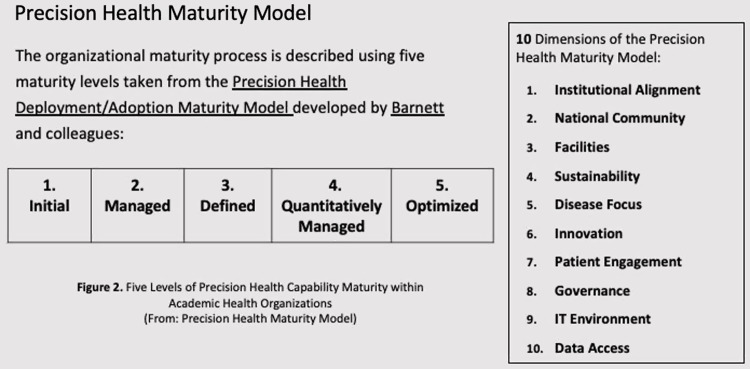
Summary of the Barnett Precision Health Maturity Model [3,5].

Within the translational science literature, learning from real-world patient outcomes is recognized as a critical strategy for moving healthcare forward [1]. Learning health systems (LHS) develop the infrastructure and capacity to systematically collect data from clinical practice so as to generate knowledge that can be applied to improve practice. Learning health systems enact “learning loops” that capture and assemble data, analyze the data to turn it into knowledge (data-to-knowledge [D2K]), build interventions that apply new knowledge to informing practice (knowledge-to-practice [K2P]), and then re-capture outcomes data to determine impacts and continue the cycle (P2D) (See Fig. 1) [2]. The sociotechnical infrastructure needed to support a mature learning process includes many of the elements of maturity proposed in the Barnett Precision Health Maturity Model, including tools and technology, human resources with operational and analytic expertise, and policies(See Fig. 2 for model summary) [3]. This sociotechnical infrastructure can exist at multiple scales, from a small group of researchers to a healthcare institution to a national network of institutions, that co-create LHS infrastructure [4].

We can characterize the maturity of an LHS system at any level of scale by observing to what extent it is able to “close the loop” across multiple processes needing improvement [6]. In this study, we examine precision oncology (PO) programs as a useful case study example of an LHS that can help us understand how LHS capabilities mature as the field attempts to translate complex, rapidly emerging knowledge systematically into practice. Figure 1 illustrates the idea that a well-developed LHS system that routinely closes learning loops will also be more mature.

This study primarily focused on the molecular tumor board (MTB) as one of the key mechanisms within a precision oncology LHS. Through stakeholder interviews with multidisciplinary professionals from academic medical centers (AMCs) and the commercial precision-medicine industry whose expertise supports MTBs, we observed the current state of maturity of the organizational routines and technologies that support the implementation of PO innovations such as genomic profiling and targeted therapeutics into clinical practice. In this paper, we identify the salient indicators of maturity rooted in LHS thinking that stakeholders use to advance the maturity of organization-driven learning within their institutions and the field of PO.

Precision oncology targets therapy at actionable mutations identified in an individual tumor’s genetic profile. PO within institutions relies on developing a learning process resembling a sociotechnical LHS system that combines expert personnel with an IT ecosystem that supports data, analytics, and systems integrations. These capacities can be realized through partnerships between healthcare institutions and commercial entities that provide services including genomic sequencing, clinical trial matching, expert support, data capabilities, and a range of MTB support offerings. As implementing PO requires significant resources, infrastructure, and expertise, different institutions adopt PO at different rates and dissimilar levels of sophistication. Therefore, we can compare the maturity of PO programs across healthcare institutions as well as consider what mature PO looks like. One way to do this is to develop a maturity model, a tool widely used and applied in software engineering and more recently healthcare [7], which can capture stages of combined infrastructure and programmatic maturity to help institutions evaluate their gaps, compare themselves to others in a competitive landscape, and identify priority areas for development to advance their maturity [8–10]. The analytic framework for the present study is to consider the current state of maturity in different healthcare institutions in terms of LHS, the impact of the critical environment on their maturity, and future strategies to advance maturity in the field of PO.

**Figure 3.**
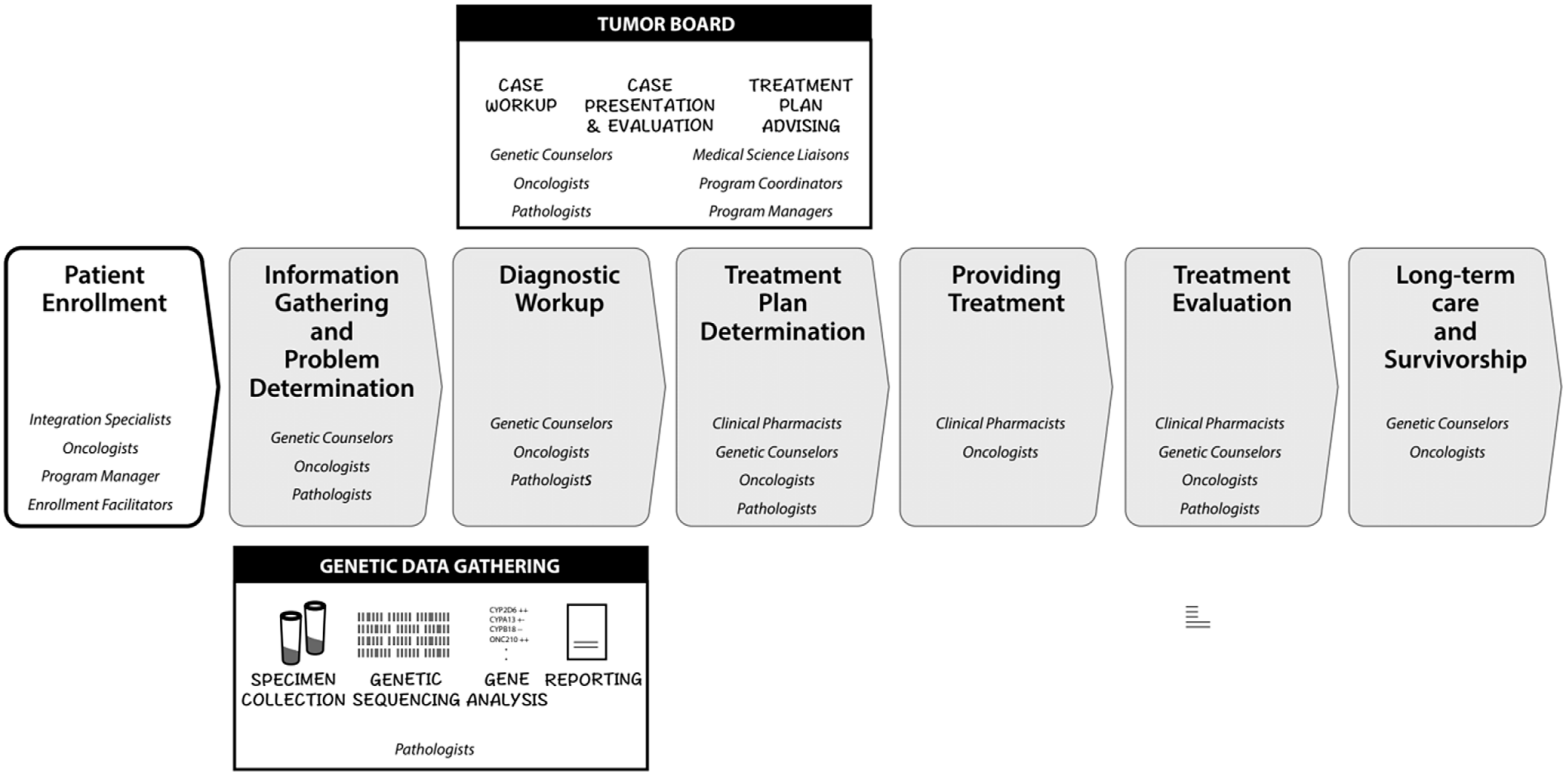
A basic and iterative genetic data collection and precision health patient care workflow.

To identify the key indicators of PO maturity, we sought to sharpen our understanding of the processes and expertise involved in daily enactment of PO. Figure 3 depicts the typical PO process of gathering information from individual patients, interpreting the information, and storing it for future use. Patient genomic information may be used during diagnostic workups to help diagnose disease, select treatment, and evaluate treatment outcomes. Using genomic data in PO practice represents the K2P portion of the learning cycle, where knowledge from public databases and multidisciplinary knowledge is applied to individual patient cases. At the aggregate level, the data collected from an individual patient can be cross-referenced with genomic warehouse data to monitor disease outcomes. LHS that take advantage of aggregate information could potentially extend across institutions to improve clinical decision-making in cases with similar genomic characteristics, which would represent the P2D stage of the learning cycle where practice is captured as data that then becomes new knowledge (D2K). Maturity models may be an avenue for comparing and coordinating the development of interoperable LHS across institutions so as to systematically capture the complex practice of PO and generate patient outcomes data that can inform learning strategies to advance the maturity of PO.

In this study, we aimed to assess the social and technical processes that define the current state of maturity in the field of PO. Four research questions guided the development of the study protocol and analysis: (1) How do people in the precision oncology ecosystem define and understand its maturity? (2) What are the barriers and facilitators to achieving maturity and how does this vary by institution? (3) What is the state of informatics and tools used within precision oncology in practice? And (4) Where should precision oncology go to move toward the next phase of maturity as a field? We also looked at how these goals could differ for AMCs and precision-medicine companies. Our analysis focused on describing the current state of sociotechnical maturity and the critical environment impacting field-wide maturity. We then considered participants’ perspectives on future directions of the field toward achieving greater maturity.

## Methods

This study was approved by the Institutional Review Board at the University of Michigan Medical Center (IRB Protocol #HUM00196813).

### Participants

We conducted 34 semi-structured 1-hour interviews with professionals serving in various roles supporting MTBs (Table 1). Interviewees were recruited through outreach at three large AMCs located in the Midwestern and Western U.S. Other interviewees were recruited within one private precision-medicine company, as well as through snowball sampling [11]. The AMCs are referred to as AMC1, 2, or 3 and the private company is referred to as Industry1. The AMCs in our study were selected as leaders in oncology and translational science. All three have CTSI awards, suggesting relatively mature institutions taking on the relatively new domain of precision oncology. These AMCs are generally well-resourced, even if specific programs have varied resources. The interviewees from Industry1 were able to comment on different levels of resource allocation with PO programs given their perspective on the market. See the basic characteristics of the AMCs in Table 1. Data were derived from AMC’s Comprehensive Cancer Center website.

**Table 1.** Characteristics of participating academic medical centers (AMCs)

Demographics	AMC1	AMC2	AMC3
Emergency Department Visits	111k+	40k+	70k+
Outpatient Oncology Visits	140k+	100k+	18k+
Oncology Faculty Members	500+	500+	300+
Associated with National Cancer Institute (have designated Comprehensive Cancer Center)	Yes	Yes	Yes
Pediatric Care	Yes	Yes	Yes
Oncology Clinical Trials	350+	250+	300+
City Population	121k+	815k+	2.6M+

Approximately one-third of interviewees were oncologists working at AMCs or in industry (*n* = 12/34). The other two-thirds of interviewees held 10 other roles supporting MTBs. Half of the interviewees held roles common to both AMCs and industry. See Table 2 for a breakdown of roles held by the interviewees.

**Table 2.** Roles of interview participants by institution group

Roles	AMC^*^	Industry	Total
**In academic medical centers and industry**			
*Genetic Counselor/Clinician*	1	1	2
*Oncologist*	10	2	12
*Researcher Scientist*	2	1	3
**In academic medical centers only**			
*Clinical Pharmacist*	1	0	1
*Program Coordinator*	2	0	2
**In industry only**			
*Clinical Trial Matching Specialist*	0	3	3
*Pathologist*	0	2	2
*Medical Science Liaison*	0	2	2
*Sales and Marketing Specialist*	0	3	3
*Technical Integration Specialist*	0	3	3
*In-house Attorney*	0	1	1
**Total**	16	18	34

* Academic medical center.

### Data Collection

The semi-structured interviews with professionals involved in MTBs occurred between July 2021 and April 2022. These interviews consisted of open-ended questions about the following topics: participant’s role within their organization and relationship with MTBs, processes surrounding MTB meetings, tools and technologies used to support decision-making, data and information technology governance, and evaluative questions about the maturity of precision oncology. Interviews were conducted by a research team member over Zoom and were recorded and professionally transcribed. Participant recruitment continued until theoretical closure was reached around key indicators of maturity, facilitators and barriers to maturity, and perceptions of future directions of PO. Theoretical closure is the process of reaching a point where primary the research topics have been thoroughly explored and where interviewers are repeatedly hearing about what they already know from other interviews [12,13].

### Analytic Framework and Data Analysis

After an in-depth review of a small sample of interviews, two research team members developed a codebook of themes related to the four primary research questions. These team members trained two other research assistants, which involved reviewing the codes of five interviews over several sessions. Once the team established consistency in coding, the full set of interviews was coded using thematic analysis by at least two team members.

**Figure 4.**
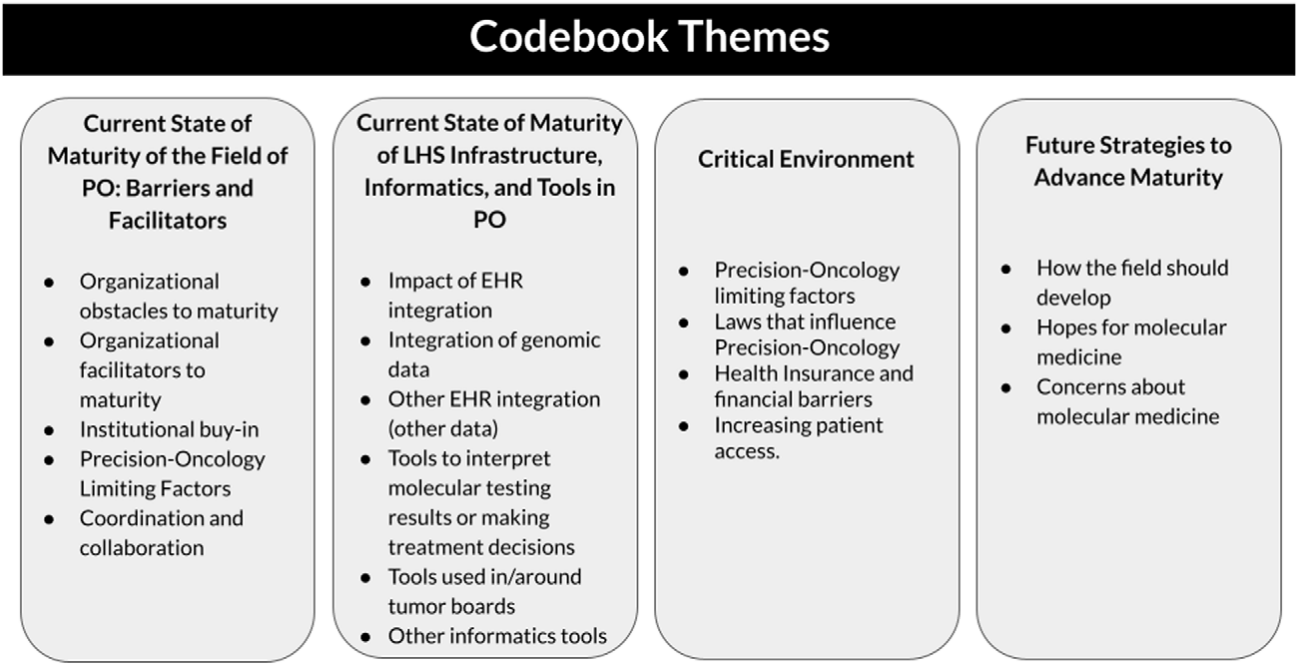
Sample codes by result themes. PO = precision oncology; EHR = electronic health record.

The codes were organized to describe current social, organizational, and technical issues in the maturity and development of the MTB as a learning mechanism. The structure of the analytical framework consisted of four domains: (1) the **current state of precision oncology** which involves barriers and facilitators to achieving maturity such as knowledge gaps, a lack of infrastructure, and institutional support; (2) the **maturity of LHS infrastructure in PO** which indicates tools for learning from practice; (3) **critical environment,** factors i.e., the policies, reimbursement, and access issues that impact maturity of the field; and (4) **future directions** for the field of PO to advance maturity. See Figure 4 for sample codes by theme and Appendix A for the interview protocol.

## Results

### Overview

The following results shed light on the current state of maturity within the field of PO, the maturity of tooling and informatics within the field, the impacts of the critical environment on field-wide maturity, and future strategies to advance maturity within PO. We found that maturity is relatively low, but continuing to evolve, across these dimensions due to the resource-intensive and complex sociotechnical infrastructure required to advance maturity of the field and to fully close learning loops.

Maturity is typically hindered by major knowledge and expertise gaps throughout the field, as well as challenges in maintaining sufficient institutional support and resources for PO program development. The tools and informatics that currently support PO implementation are usually basic and often locally developed. More advanced tools and learning capacity development are driven by precision-medicine companies. Institutions are particularly strong in incorporating new K2P for learning purposes by utilizing publicly available databases to inform PO treatment decisions. However, institutions struggle to extract lessons from every case for potential future application to similar cases. The critical environment impacts the maturity of the field through legal and insurance policies that slow the sharing of data and patient access to PO services.

**Figure 5.**
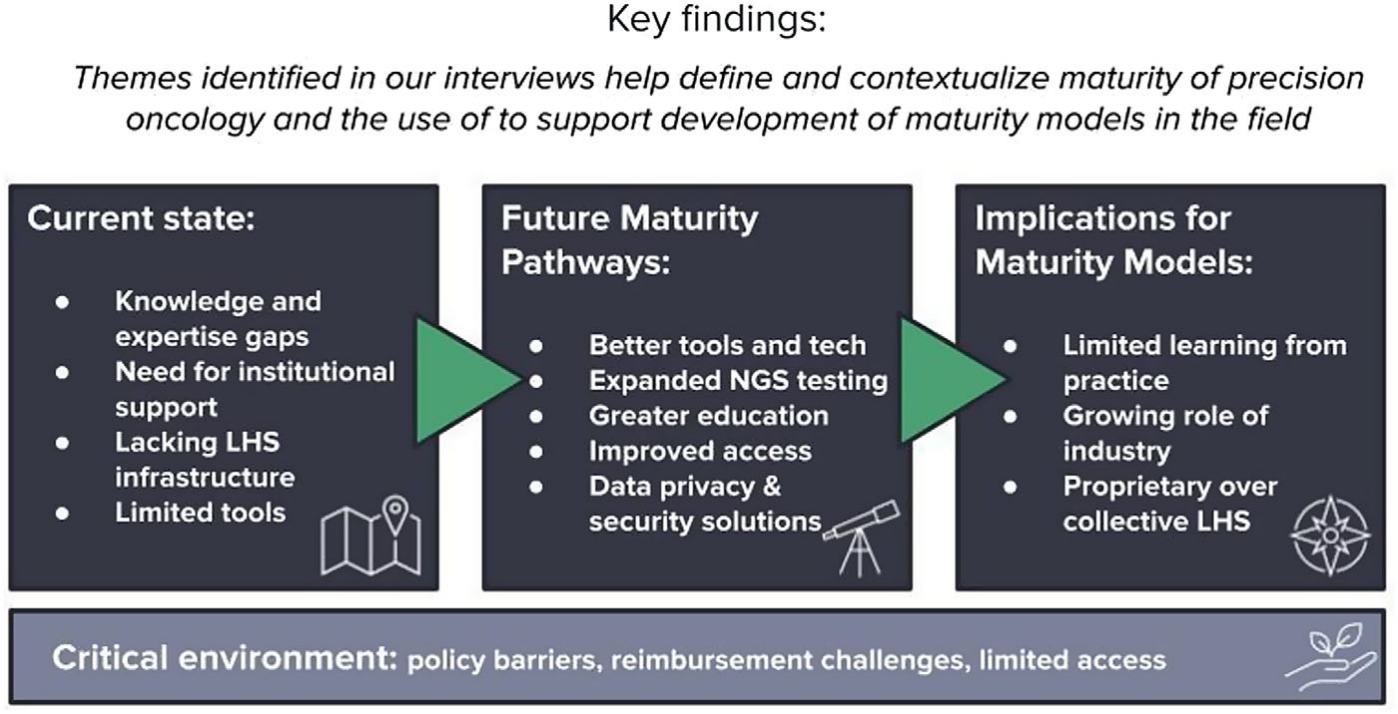
Overview of key findings. LHS = learning health system; NGS = next generation sequencing.

Interviewees had many ideas of how the field will continue to evolve and advance toward maturity, including more technologies being developed to improve preventative and targeted treatment options within PO, greater education of stakeholders can expand the practice of PO, and development of new governing mechanisms to keep patient data safe will enable ethical learning. Figure 5 depicts these key findings that inform our overview.

### Current State of Maturity of the Field of PO: Barriers and Facilitators

Despite rapid scientific innovation and interest in expanding the practice of PO, we found that there are significant barriers to achieving institutional and field-wide maturity in PO. First, results revealed that many physicians and other professionals lack the advanced cancer genetics knowledge required to readily apply PO in practice. As one participant from Industry1 articulated,

*“That level of complexity just for one patient, […] it’s very different than it was […] five years ago, there was not this level of genomic complexity, and so this is really … It’s a lot to contend with. The science is just evolving so fast and there’s very little consistent definition in the drug development space.” Clinical Trial Matching Specialist, Industry1.*

It is challenging for clinicians to keep up with the rapid evolution of science and available PO therapies, leading to a mismatch between social and technological capacity. An Industry1 Pathologist described factors thought to contribute to the knowledge and expertise gap, such as whether physicians practice in an AMC or community oncology and the heterogeneity of when physicians were trained. The knowledge gap can range from basic challenges around choosing the appropriate genetic diagnostic test to interpreting the test results (see quote 1, Appendix B). This is echoed by an oncologist at AMC2 who runs their MTB and describes the educational function the MTB plays in helping other oncologists who have less subject knowledge and training to determine PO treatment options (see quote 2, Appendix B). MTBs help fill knowledge gaps through multidisciplinary discussions to collaboratively determine treatment plans for patients with genomically complex cancers. Industry1 participants described administrative and expert support they provide through MTB services with community and AMC partners, although some interviewees predicted a move toward multi-institution MTBs due to limited capacity to support upwards of 20 MTBs across partner institutions. Many AMCs rely on companies like Industry1 for services, especially those who have not developed internal NGS services and do not have in-house expertise (see quotes 3, 4 Appendix B). The maturity of the field of PO is limited by the availability of expertise, which is challenging to grow and scale quickly.

Multiple stakeholders tied maturity to having the institutional support to create robust, systematic PO programs. They named certain well-known, well-resourced cancer centers as points of comparison. These institutions had devoted resources to sequence and consider every cancer patient for PO therapies regardless of cancer stage, creating a more systematic institutional approach centering PO as a core part of cancer treatment. AMC interviewees described how their PO programs rely on financial investment and support from their institutions to build and maintain the expertise and infrastructure to improve their programs. Interviewees from Industry1 often commented on the variation in how much institutions support developing robust PO programs and in the approaches to determining which patients to sequence, observing, “*If you’ve seen one cancer center, you’ve seen one cancer center”- Sales and Marketing Specialist, Industry1*. While some institutions systematically sequence all patients, other PO programs might only encourage sequencing for select cancer patients or rely on physician discretion about when to sequence. Additionally, we found examples of individual champions within PO programs who were successful in garnering institutional support but left the programs vulnerable to leadership changes. For example, one participant expressed the challenges of losing an individual champion along with the institutional support to hire the appropriate expert contributors to strengthen their PO program moving forward:

*“We just lost [former PO champion leader]. And we have to find some interim director but the rest of the people are still here, but unless we suddenly find some serious amount of institutional support, including our ability to recruit and retain people for specific purposes, I do not think anything is going to change. I know it probably sounds pessimistic, but in other words, it takes a village to do this kind of stuff.” - Research Scientist, AMC3*

Another participant echoed the importance of an institutional strategy that brings together the right types of expertise to develop a mature PO program, commenting:

*“I think that AMC1 approach to precision oncology has been haphazard and has not invited the key stakeholders to the table. So, for instance, AMC1 precision oncology initiative never invited any of the clinical geneticists to any of the planning meetings, and the fact that it was run by anesthesia and by biostatistics seemed like a big problem because they later found that they needed epidemiologists to be able to identify why their recruiting pattern led to bias…” - Genetic Counselor/Clinician, AMC1*

This highlights the wide range of expertise and buy-in from many stakeholders needed to develop a comprehensive, robust, and cohesive PO program, which must be facilitated at an institutional level to ensure proper stakeholders are engaged at key decision points.

The AMCs and Industry1 have developed strategies that facilitate the advancement of maturity through learning mechanisms. For example, some AMCs have a pathology team that conducts literature searches compiling the latest information about mutations and potential targeted therapies for patients. Expert-run literature searches presented during MTB meetings aimed to bridge existing aggregate field knowledge to the individual case to determine the best course of action. A pathologist explains:

*“We had a curated literature database of all the drugs that had previously been assessed. But what we would do is every time we got a new patient, we would evaluate the literature for any new findings about that mutation, because sometimes there would be new studies to show that the mutation was actually a resistance mutation to a medication, rather than a sensitive mutation, and so that might change our recommendation. And then we would also do a literature evaluation for any drugs that targeted that mutation, to see if there was new data to show penetration, if there were any new agents in the pipeline. And so we would essentially get two or three cases, usually no more than four cases that were presented at each tumor board, and do a very thorough literature review of what the potential options were, and then come up with a recommendation after we collated all of that information or data.” - Pathologist, AMC2.*

This demonstrates that PO learning processes are resource-intensive, in that they often require significant expertise, manual effort, and time in order to curate the best treatment plan for each patient.

### Current State of Maturity of LHS Infrastructure, Informatics, and Tools in PO

Another indicator of maturity is the state of tools and informatics within the PO field. From an LHS perspective, having appropriate tooling and infrastructure for systematic learning would indicate greater maturity. However, while PO is a highly innovative space, much of the everyday practice of PO is supported by relatively basic, low-tech tools, such as PDF reports for genetic sequencing results and PowerPoint slides populated with report screenshots used during MTB discussions. Some interviewees considered implementing commercial or locally developed tools to improve their workflows and learning capacities, such as project management tools and databases to help track cases, recommendations, and outcomes. Although some interviewees were aware of commercial PO tools, participants reported that workflow management and systematic learning from MTB discussions is still an ongoing problem they would try to address with their own basic tools. For example, an MTB program coordinator at AMC2 described creating their own slide libraries, MTB triage tools, and recommendation databases to reduce work and make decisions more consistent across patients at their institution (see quotes 5, 6 Appendix B). They also expressed having limited resources to build and manage these tools. Other AMCs appeared to be farther along in development of internal databases for tracking sequencing results in a way that could support clinical improvements. An interviewee from the AMC1 explains,

*“We actually have a separate database…where we can track all of our patients that have been sent for genetic testing and we can track what they were tested for and what the results were. […] If we see a variant, we can put in the variant and find who else we’ve tested who had a germline alteration that had the same variant. That way, we’ve been able to connect families. ”- Genetic Counselor/Clinician, AMC1.*

Although both AMC1 and AMC2 conduct NGS locally, they describe different levels of internal database development and support. However, both sought to systematically learn from previous cases to inform other cases, suggesting progress toward building LHS for PO. While tools and databases like these can improve systematic application of PO within one institution if appropriately resourced, they fall short of addressing the potential lack of consistency in PO offerings across institutions and beyond.

While localized tools enabled some institutional learning from MTB decisions, there was less evidence of building broader, multi-institutional learning mechanisms to advance field-wide maturity. Given Industry1’s role in providing NGS services to many AMCs that lack the infrastructure and facilities for genomic sequencing, they are now stewards of genomic data from many institutions. To leverage these data for future cases, Industry1 interviewees described efforts to support learning by creating a user-friendly tool that allows clinicians to explore research or clinical questions, such as comparing a specific patient case to a larger dataset to understand alteration prevalence. Industry1 has also created clinical trial matching programs for patients who have undergone NGS. These programs aim to find rare patients who could benefit from active clinical trials and either alert their physician to a nearby trial or rapidly initiate the trial on-site for an eligible patient when possible through a proprietary partnership with the trial sponsor (see quote 7, Appendix B).

Industry participants also discussed the development of other tools and services that could potentially improve workflows, data quality, and learning within PO. For example, PDF reports are the most common format for delivering sequencing results and are often manually scanned into local EHRs. To address this, Industry1 is building genetic data integration pipelines that move PDFs and discrete data directly into EHRs. An interviewee from Industry1 reported,

*“We’re providing back PDFs so their EMR could facilitate a tumor board that way, where they just create patients onto a list, go through the list and pull up the medical record, click on the PDF in the medical record. ”- Technical Integration Specialist, Industry1.*

As these services are established with more healthcare institutions, precision-medicine companies are playing a greater role in advancing field-wide maturity by contributing to the development of shared and interoperable LHS infrastructure.

Institutions heavily rely on integrating various databases, including internally developed and public databases, to support their practice of PO. AMC1 uses public databases, including the ClinVar, to support their NGS sequencing results report recommendations with relevant literature and data to support drug and genetic categorization. ClinVar is a public database that serves as a resource for clinicians and researchers who want to understand genetic variations and their impacts on health [14].

*“…Based on the criteria of the amount of data and the strength of the data, laboratories make a judgment call as to where it falls on that spectrum. It’s a variant of uncertain significance, it’s right in the middle. If it’s likely pathogenic or likely benign, we will often look at those data and actually look at ClinVar to see how many labs have classified the variant and how the variant has been classified by different labs. So, for instance, if there’s a variant that’s been classified as pathogenic by one lab but every other lab classifies it as variant of uncertain significance, we’ll talk about the strength of the data and what we think might be going on.” Genetic Counselor/Clinician, AMC1.*

This demonstrates how publicly available information infrastructure allows healthcare institutions to create robust processes for integrating new knowledge into practice as part of the learning cycle (K2P). Industry1 also integrates this information and clinical trial matches from clinicaltrials.gov into NGS sequencing reports. While this part of broadly accessible LHS infrastructure is the most well-developed, the D2K and K2P portions of the cycle come largely from clinical trials and not from real-world evidence based on PO practice at the local or broader scale.

### Critical Environment

Interviewees also recognized challenges and critical elements in the current environment as pervasive and limiting to advancing maturity. First, while the Health Insurance Portability and Accountability Act (HIPAA) enables patient data sharing and interoperability, its provisions are cumbersome and outdated for PO practice. Some participants from AMC1 and Industry1 report that HIPAA requires large amounts of genomic data to be protected in ways that institutions struggle to manage. Additionally, HIPAA’s different approaches toward patient confidentiality between clinical and research contexts can make it confusing to discuss certain patient cases, while aggregating sufficient quantities of de-identified data for sharing and learning purposes can take a long time due to the rarity of certain mutations (see quotes 8, 9, 10, 11 Appendix B). Second, sequencing technology outpaces insurance company reimbursement. Some payers allow individual gene code stacking to bill for NGS instead of creating standardized codes for NGS tests. One participant from Industry1 reports, “*some payers like Insurance Payer 1 for example, have put in a cap, you can only bill for genes. And, in other situations, you do not*” *(Sales and Marketing Specialist*). Third, the cost of PO can be high, limiting access and potentially making health disparities worse. Many participants from AMCs and industry shared concerns about the cost and accessibility of PO. An AMC2 participant said, *“I worry that so many of these ideas get kind of pushed into a commercial platform that then is very costly and limits access to many people”(Research Scientist).* A participant on the commercial side shared similar worries: *“My concern is cost and access. I mean, people of color, they’re not even getting tested at all and they’re not represented in our genome atlas” (Clinical Trial Matching Specialist, Industry1).* These discussions should be placed in context of the limitations of how we currently fund healthcare and how to balance resources across the population.

### Future Strategies to Advance Maturity:

We asked interviewees to imagine what the future of maturity could look like for PO as suggestions for further maturity optimization. Participants offered tangible ways that emerging technologies could increase the maturity of practices within PO. For instance, one interviewee described medically ambiguous situations today that could be clarified through improved techniques to distinguish between potential therapy options for a given patient. They explained,

*“‘Do I give my patient MEK inhibitor versus PIK3CA inhibitor?’ Well, that could be a tumor board question today, but in the future, we could be doing, organoid drug profiling or maybe there’s some predictive algorithm using gene expression that can give you some type of differential response prediction where they’re like, “Oh, this patient clearly would respond to MEK and MEK inhibitor.” And so, that would be something where maybe technology solves an ambiguous situation today.” - Pathologist, Industry1.*

As technology becomes more mature, the role of the MTB could prioritize the more ambiguous, complex cases and rely more on clinical decision-support tools to guide the more straightforward cases. Interviewees also touched on the possibility of innovations enabling preventive forms of PO care, where insurance could potentially cover genomic sequencing tests to proactively identify high-risk patients (see quote 12, Appendix B).

As PO continues to evolve rapidly, interviewees recognized a growing need for more education to empower clinicians and patients to navigate the expanding landscape of testing and treatment possibilities while maintaining reasonable expectations for what PO is capable of achieving (see quote 13, Appendix B). Additionally, interviewees raised concerns about the privacy and security of patient data, as the expansion of PO will require increasing amounts of sensitive genomic information to be collected and governed. As one participant emphasized,

*“And also, privacy is a huge thing. The more tuned in and the more we’re able to kind of identify all the issues, identify all these mutations, the more we’re able to, without giving PHI, giving names, be able to still identify these patients from their biological signatures. So it’s kind of a double-edged sword, we’re finding out a lot more about people through precision medicine but with that comes the responsibility of being able to keep that information private and not exploited in any way. ” - Program Coordinator, AMC1.*

Stakeholders acknowledged that the critical environment will have to respond to these issues by developing policies, strategies, and infrastructure for learning from this data systematically while protecting individual patients’ right to privacy.

## Discussion

This study illuminates social learning processes in the context of advancing maturity of precision oncology. Through the combined lenses of LHS and maturity models, we are able to consider both the social and technical dimensions underlying the development of emerging fields within healthcare. Through stakeholder interviews with interdisciplinary MTB team members, we assessed the current state of maturity of the PO field. Our preliminary work demonstrates that PO is a useful case study of developing an LHS, where emerging technologies and innovations require new sociotechnical processes and routines to realize and apply new learning. Participants assessed the maturity of their PO programs, allowing for comparison of internal facilities, tooling and capabilities, institutional investment, and rate of PO adoption across comparable AMCs. The most well-established part of the learning loop in the PO field is in the D2K part of the loop where clinical trial results are routinely incorporated into publicly available databases that regularly inform practice. However, closing the learning loop by capturing performance (P2D) is not yet routine due to the challenges of collecting the data needed to evaluate the real-world outcomes of PO approaches. Although not always systematic, MTBs served as a primary mechanism for integrating multidisciplinary expertise and applying field-wide knowledge to individual patient cases. However, the field of PO is not yet systematically learning from practice to improve performance.

Participants described barriers that made it difficult to close the learning loop at scale in order to “learn from every patient” [1]. Proprietary, bespoke tools at individual institutions were more common than investments in larger learning infrastructures. For example, programs used simple strategies such as MTB PowerPoint slide libraries and treatment recommendations databases that could be used to compare similar cases. However, they reported limited resources to support building or purchasing existing tools for learning from practice. Given these limitations, commercial entities (including precision-medicine companies) fill the role of creating interoperable LHS infrastructures across PO programs as opposed to broadly accessible public goods.

**Figure 6.**
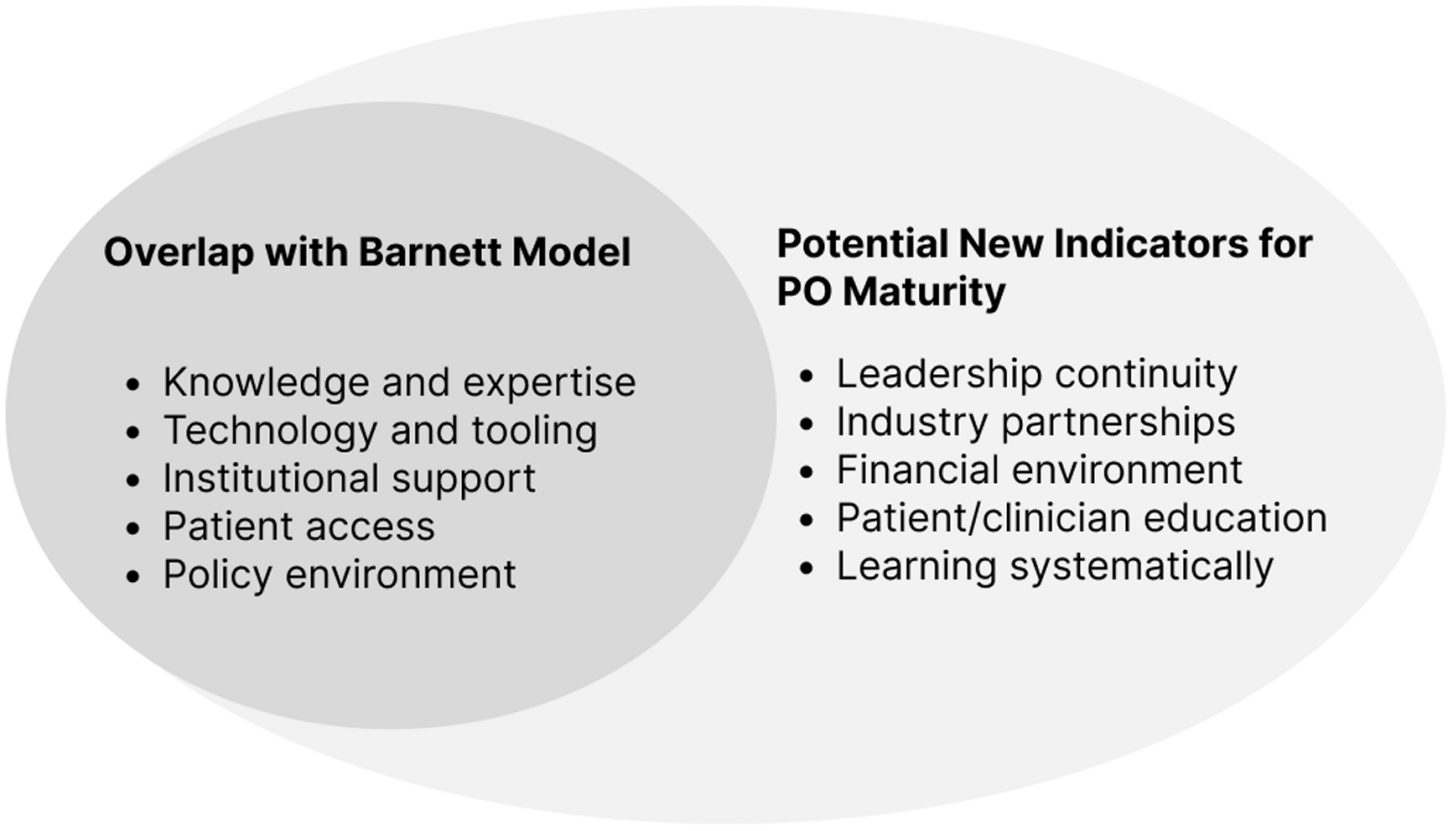
Potential indicators of maturity for precision oncology [5]. PO = precision oncology.

This study is a window into the current state of the practice of precision oncology, identifying a set of indicators of maturity that could inform future iterations of maturity models. Figure 6 shows potential indicators that could be incorporated into subsequent maturity assessments.

Participants raised issues that affect both the maturity of the institutional context and the field, including knowledge and expertise gaps, institutional support, lack of LHS infrastructure, and limited tools. By identifying the processes and people involved in PO, we move toward a better understanding of how organizations could move from one maturity level to the next. While other studies have looked at case examples of implementation of PO from the healthcare delivery point of view [15], we were able to show how industry interacts with AMCs to enact PO and demonstrate its role in driving field-wide maturity by providing expertise and sophisticated tools. Understanding participants’ expectations for the future of PO creates meaningful goalposts for achieving the next stage of field-wide maturity, such as improving tools and technology, treatment options, patient and physician education, and access. Additionally, we have connected the concept of maturity to the framework of LHS, which can inform the development of maturity strategies that aim to close the learning loop in order to move the field, as well as other translational science contexts, forward. Maturity strategies can also be applied by PO program management to improve program sustainability by offering a means to prioritize program investment while relying less on individual champions.

The existing translational science literature recognizes that rapid learning from real-world patient experiences with PO is an important strategy for moving the field forward [1]. However, outcomes of MTB decisions and other clinical PO decisions have only been studied on a small scale, which limits learning [16,17] from practice as our work suggests. Other scholars have noted similar challenges in implementing PO, such as lacking resources, infrastructure, education, and sufficient decision-support tools [18,19]. Furthermore, informatics tools tend to be developed internally by the most well-resourced organizations. Studies have noted similar constraints in the critical environment, such as inconsistent reimbursement for genomic testing and limited targeted therapy access [15]. We support these previously identified concerns with real-world examples from our PO stakeholder perspectives, including from the industry point of view. Finally, others have developed maturity models as self-assessment tools for multidisciplinary teams for cancer care to inform performance improvement over time [20]. We extend maturity model utility by conceptualizing the indicators of maturity in a precision oncology context that could drive LHS maturity strategies in an emerging field and other translational settings (See Fig. 6).

This study has limitations that are important to consider. We interviewed a modest number of participants who held various roles at only four research sites, which included industry and AMCs. The findings may not be generalizable across all practices of PO, particularly for community oncology practices which generally have fewer resources and may have different capacities for achieving maturity. However, we believe that most research sites will report similar issues; further work will be required to determine this including larger multi-site studies for the creation of a PO maturity model.

Future studies should focus on how PO as a whole may be at risk for worsening existing health disparities through differential levels of access to these emerging technologies. It would require a wider group of stakeholders and additional interview questions to elicit perspectives on how institutions are addressing health disparities within PO. Future research should also examine how variability in the systematic application of precision-medicine impacts the maturity of the field of PO, and consider the use and applicability of maturity models as standard measures of maturity. Further work should explore the ways in which the critical environment is a barrier or facilitator to attaining field-wide maturity, capturing factors such as data privacy laws and regulations, insurance coverage policies, and equitable access to PO services. Finally, this study can inform the development of future maturity models to guide the practical implementation of precision oncology programs and other translational science applications.

## Supporting information

Hoffman-Peterson et al. supplementary material Hoffman-Peterson et al. supplementary material

## Supplementary material

The supplementary material for this article can be found at https://doi.org/10.1017/cts.2023.682.
