# Supplementary material for: Advancing maturity modeling for precision oncology
Source: J Clin Transl Sci. 2023 Dec 7;8(1):e5. doi: 10.1017/cts.2023.682 (PMC10879851; doi:10.1017/cts.2023.682)
Supplement: Hoffman-Peterson et al. supplementary material [file S2059866123006829sup001.docx]

**Appendix A: Interview Protocol**

**Molecular tumor board participants**:

- Clinicians: oncologists, pathologists, surgeons, radiologists, radiotherapists
- Scientists: molecular biologists, geneticists, biostatisticians, computational biologists, bioinformaticians
- At times, genetic counselors and pharmacists
- Study coordinators
- Other administrative/support staff

**Interview duration:** 45-60 minutes

**Questions about the participant:**

1. What is your role in the organization?
2. How long have you worked in this organization?
3. What is your role in the molecular tumor board?

**Process-based questions:**

1. Describe the people involved in precision oncology at your medical center.
2. What role do molecular tumor boards play in cancer care at your medical center?
3. Describe or map the processes leading up to meetings of the MTB. (global vision)
   1. Among these processes, which ones are the most important or critical? (ask about the most critical and then go down the list)
4. We are interested in understanding the people, processes, systems, and milestones that interface with decision-making in precision oncology. Please pick a recent case, and lead me through the treatment trajectory, starting from patient consent and biopsy to the disclosure of results.
5. How typical is this case? If yes, might answer 3 partly
6. (What makes this case typical?)
7. If not, ask for a more typical case
8. Pick a recent atypical case, explain what was different, and how it was handled.
9. How do you and your colleagues learn and apply lessons from one case to future cases?
10. What are examples of policies used to govern and develop precision oncology at your organization?
11. How are patient cases selected and prioritized for review by your center's MTB?
12. How do clinicians/you decide what genetic testing to order, from which lab, when in the disease course, and using what biomaterial?
13. What tools do clinicians/you use in interpreting test results and making treatment decisions?
14. How and when are patients informed (about what?) along the course of the treatment trajectory?

**Data and Information Technology:**

1. Where is genomic testing data stored at your medical center?
2. Who owns/controls the data?
3. Who maintains it?
4. Who governs storage and capacity?
5. How are clinical and genomic data integrated in your organization's EHRs or other systems?
6. Do you use any new tools or specialized software systems specifically to support tumor diagnostics or precision medicine?
7. If yes, what new tools or systems do you use?
8. What are your opinions about the use of these new tools or systems?

**Evaluative (organization and ethics):**

1. What do you think about the way your organization approaches precision oncology? How would you say your organization is doing, and where would you like it to go?
2. In what ways are patients and community stakeholders involved in precision oncology at your organization? What is the purpose of patient and community engagement and do you achieve your objectives? In what ways are you successful? What might be improved?
3. In five years, what do you think will be different about the MTB?
4. What are your hopes and concerns about molecular medicine and its implications for patients and/or populations?

**Closing question**: Is there anything more you would like to add?

**Appendix B: Quotes Referenced in Text**

| **Theme** | **No.** | **Quotes** |
| --- | --- | --- |
| **Current State of Maturity of the Field of PO: Barriers and Facilitators** |  | *“There's a lot of heterogeneity between people in different, I would say, you could kind of break it up into academia versus the community, and also there's heterogeneity between when you did your training. And so, people that are younger, obviously, are more well-versed in molecular biology versus people that are older are going to be less well-versed in it. And so, I think that's probably a big driver of the need for the molecular tumor boards is you have variation of where you might have a tumor board where literally the oncologist kind of, A, they're not going to know the difference between what a PCR test and an NGS test is. And that can be a big deal because it's like PCR tests, in general, you're kind of testing for one gene at a time, you're doing these sort of a la carte gene testing versus NGS, you're doing simultaneous testing of all these different genes. And so, even just understanding those kinds of basic things may not be clear sometimes. And also, sort of like what an alteration means can be sometimes unclear. So if people don't have a great handle on how to interpret reports or sort of like what the results mean, you could have people misunderstand what the significance of a molecular result is and they might put a patient on a drug or a clinical trial incorrectly.” - Pathologist, Industry1* |
|  |  | *“I think there's a couple of components. One is education because a lot of our oncologists, this is a brand new kind of world of medicine. They haven't had any training in genetics or how next generation sequencing works and what are the limitations of that assay, and how to interpret that information. What does a wheel fraction really mean? What's the difference between somatic and germline, things like that. So there's absolutely an education component that goes into it. I would say the vast majority of the people coming to molecular tumor board are looking for genomically driven treatment options or genomically driven clinical trial options. So they're really asking our experts to provide treatment insights based on the molecular profile of the patient's tumor.” - Oncologist, AMC2.* |
|  |  | *“We're there to answer questions that doctors have about the report to help understand. So that could be about the type of assays that we have, how things that are detected, explaining different phenomena, like the VIF being really high and compared to what's expected like these kinds of things, and then explaining mechanisms like pathways that are activated, explaining how therapies target those pathways.” - Research Scientist, Industry1.* |
|  |  | *“ ….So the patients undergo tumor biopsies, and the samples; the DNA, RNA, they're sequenced by sequencing companies, either on site or by third-party companies. Then we have the sequencing data back based on both DNA sequencing, RNA sequencing, and the mutational data versus sequencing the data. Then most of the clinicians, even at some of the nation's top biomedical institutes or cancer centers, these practicing clinicians are not too well-equipped to dissect biological information, especially beyond what they know about, which is very limited, because their job is to treat patients. So their job is not necessarily to understand the biological underpinning of what they're seeing. So that's where we try to meet somewhere in between. …” - Research Scientist, AMC3* |
| **Current State of Maturity of LHS Infrastructure, Informatics, and Tools in PO** |  | *“With the recommendations database, I've long wanted to kind of create a library of around that, because very often, I'm recycling the same paragraph, the same text, and I'm spending a lot of time hunting down that recommendation report where I discuss that particular finding that I know I want to pull forward into this other recommendation report.” - Program Coordinator- Tumor Board, AMC2.* |
|  |  | *“We've been building what we call an MTB triage tool […] which is what are the cases that should be discussed at the Molecular Tumor Board, and what kind of priority level should there be assigned to those?” - Oncologist, AMC2.* |
|  |  | *“The tool itself has dramatically increased in its utility over the past year. And that's more of a result of us just committing more resources to the tool itself, both from a data and engineering and then user experience perspective[…]We'll come across a case where the literature may say this particular alteration in this cancer type is seen 0.07% of the time, but then to reference a data library in the millions and be able to understand that "Hey, that data does hold up." Or, "Hey, this may be less or more prevalent," that does provide a great deal of confidence.”- Sales and Marketing Specialist, Industry1.* |
| **Critical environment** |  | *“A lot of the information has to be stored on cloud servers, but one of the challenges is that the patient confidentiality has to be maintained too. So we have all of these requirements of HIPAA which are for patient confidentiality and things. So the hospital data systems is doing a lot of this. But I think this is a challenge for the future because there's going to be a strain between the tremendous amount of data that's going to be available in each patient and where is it going to be stored and how are you going to make sure that it's secure and how are you going to access too?” - Oncologist, AMC1* |
|  |  | *“It gets really complicated in terms of what you're allowed to document on patients and share with HIPAA requirements. Because some of these are research studies and so that data could be shared with investigators, but without additional approvals, you can't always share that data clearly with other individuals. And so you have to wait until you have a large enough cohort to do some sort of descriptive or statistical analysis before pushing out that information and because some of these molecular targets are so rare, that takes a really long time.” - Clinical Pharmacist, AMC1.* |
|  |  | *“So I think it's a combination of both. I think with these types of things, it gets really complicated in terms of what you're allowed to document on patients and share with HIPAA requirements. Because some of these are research studies and so that data could be shared with investigators, but without additional approvals, you can't always share that data clearly with other individuals. And so you have to wait until you have a large enough cohort to do some sort of descriptive or statistical analysis before pushing out that information and because some of these molecular targets are so rare, that takes a really long time.” - Clinical Pharmacist, AMC1.* |
|  |  | *“Industry1 can, and it does, deliver raw data back to sites that they would store themselves. The problem with that is it can be quite complex. It's not really a digestible manner, it requires the technical aspect of interpreting the data. Not to mention storage as the question revolves around, it's a safety issue and HIPAA and so on. So a lot of times, what I've seen is that sites come to the Industry1 platform, get their data, digest it, and then they don't necessarily want their raw data most of the time, because then they're responsible for it. But the raw data is available and some sites do take it. It's just, I'm not sure what they do with it.”- Oncologist, Industry1.* |
| **Future Strategies to Advance Maturity:** |  | *“There are a lot of things that could be addressed a lot sooner if we were to [...] sequence [...] hundreds of people, there might be a couple people that didn't realize that there were a very high risk of breast cancer or very high risk of heart disease and things like that, but they can be able to utilize that, hopefully with their insurance, to get better preventative care”- Program Coordinator, AMC1.* |
|  |  | *“So I think that there needs to be a push and more on the education side to really help adopt this better. And I think that's kind of the missing link. I just think that it's going to require some patient education too, for them to understand what is offered currently and what can be done.” - Medical Science Liaison, Industry1.* |
